# Host genes and their effect on the intestinal microbiome garden

**DOI:** 10.1186/s13073-014-0119-x

**Published:** 2014-12-17

**Authors:** Jonathan Jacobs, Jonathan Braun

**Affiliations:** Division of Digestive Diseases and Department of Medicine, David Geffen School of Medicine, University of California at Los Angeles, Los Angeles, CA 90095 USA; Department of Pathology and Laboratory Medicine, David Geffen School of Medicine, University of California at Los Angeles, Los Angeles, CA 90095 USA

## Abstract

We are only beginning to understand the relationship between host genetics and the gut microbiome. Two recent studies help to disentangle this interaction and show that genetic loci across the human genome shape the gut microbiome. This opens the possibility that an unexpected number of genetic factors act directly on microbial composition and function to modulate immune pathways and metabolic phenotypes in host physiology and disease.

## Association of host genetics and the microbiome

The small intestine and colon house a complex bacterial ecosystem that plays a critical role in host metabolism and immunity. Variation in the microbiome within human populations may influence susceptibility to diseases such as obesity and inflammatory bowel disease (IBD), motivating efforts to understand the determinants of the composition of the intestinal microbiome. There is extensive literature on the effects of diet, antibiotic exposure, colonization history and other environmental factors. Whether host genetics also influences the microbiome is of growing interest; two recent studies, one by Goodrich *et al*. in *Cell* [1] and the other by Knights *et al*. in *Genome Medicine* [2], have addressed this question.

It was already known that microbial composition is altered when genes that encode host mechanisms for microbial sensing and effector functions were knocked out in mouse models [3]. Other studies had also shown that genetic disruption of host microbial regulation in animals can induce susceptibility to colitis or metabolic syndrome. This susceptibility is transmissible via the microbiome, demonstrating the relevance of gene-microbe interactions to human health [4,5].

In humans, it is unclear whether genetic variation in human populations contributes to the inter-individual diversity in the human intestinal microbiome. This question can been addressed by evaluating whether the composition of the microbiome is more similar between monozygotic twin pairs than between dizygotic twin pairs, an approach that controls for shared environmental factors. Two early studies using high-throughput 16S sequencing reported no difference in microbial similarity between monozygotic twins compared with dizygotic twins using a phylogenetic similarity measure [6,7]. However, these studies had low power due to a limited number of twin pairs (54 or fewer) and global endpoints of microbial composition (for example, principal component analysis), leaving the issue open to debate.

In this issue of *Genome Medicine*, Knights *et al.* present a systematic analysis of the effect of 154 IBD-associated polymorphisms on microbial composition in three cohorts of patients with IBD (152 to 162 patients in each cohort) [2]. The authors created multivariate linear models incorporating the IBD-associated polymorphisms and various clinical metadata to predict the abundance of bacterial taxa present in at least 75% of samples. Genetic variants associated with altered taxa abundance in one cohort were then cross-compared to the other two cohorts to determine if the directionality of change was conserved across cohorts. A total of 49 IBD-associated genes, representing nearly a third of the known IBD-associated genes, affected microbial taxa in a concordant manner in at least two of the cohorts. Three Reactome pathways were enriched in these genes: innate immune response, inflammatory response and the JAK-STAT cascade. These findings are consistent with animal studies demonstrating that perturbation in immune function can greatly influence microbial composition. The six known disease-associated variants of NOD2, an intracellular sensor of bacterial products, were combined in a separate analysis. They were found to influence overall microbial composition and the abundance of Enterobacteriaceae. Although NOD2 was one of the strongest microbiome-associated genetic variants, in linear models it had a modest effect relative to clinical metadata, such as antibiotic usage. This suggests that the influence of genetics is likely to be highly specific and requires careful study design to separate it from the many environmental influences on the microbiome.

## A heritable taxon protective against obesity

These concepts are echoed in a structurally distinct study by Goodrich *et al.* in the November issue of *Cell*, which analyzed a host genomic input on fecal microbial composition, utilizing the power of twin studies (TwinsUK cohort;171 monozygotic twin pairs and 245 dizygotic twin pairs) [1]. The increased statistical power compared with earlier twin studies allowed the authors to identify a statistically significant increased similarity in microbial composition within monozygotic twin pairs compared with dizygotic twin pairs. Moreover, the effect of genetics was found to be stronger for some bacterial families than others. Among the three dominant families, Lachnospiraceae and Ruminococcaceae composition were significantly more similar within monozygotic twin pairs whereas Bacteroidaceae composition did not differ between twin types. The most highly heritable family was Christensenellaceae, which correlated strongly with several other taxa, including Methanobacteriaceae, which were also highly heritable. This network was enriched in lean individuals compared with obese individuals, suggesting the existence of a heritable consortium of bacteria that protect against obesity. Human microbial reconstitution studies using germ-free mice confirmed the predicted association of Christensenellaceae colonization with the lean phenotype.

## Genotype-microbiome interaction to disease phenotype: a complex interplay

The findings of Goodrich *et al.* suggest that heritable susceptibility to diseases such as obesity may be partially attributable to genetic factors that change the microbiome, although the specific common genetic variants in the human population that influence the microbiome remain unknown. Knights *et al.* successfully tackled this issue by identifying 49 IBD-associated polymorphisms that were correlated with microbial composition in some of their IBD cohorts. Indeed, many of the 163 known IBD genetic risk loci are involved in host-microbe interactions [8].

Existing studies of candidate IBD-associated genetic variants have identified alterations in the intestinal microbiome in individuals carrying polymorphisms in NOD2 and FUT2, an enzyme that fucosylates mucus glycoproteins [9,10]. The challenge now is to clarify if these variants contribute to disease phenotype through their direct influence on microbiome selection, whose products in turn elicit the disease phenotype. This question can be addressed by determining if genetic polymorphisms concordantly affect microbial composition in healthy individuals. Considerable further work will be required to elucidate the mechanisms underlying the observed genetic associations with microbial composition in these two studies. This will entail large-scale genome-wide association studies in both healthy individuals and individuals with microbiome-associated disease, similar to the study by Knights *et al.*, paired with validation using gene-targeted and human gnotobiotic mice. Such investigations will not only aid our understanding of human disease, but could unmask microbes such as *Christensenella minuta* that are relevant to human health and could be manipulated for therapeutic purposes.

## Abbreviation

IBD: Inflammatory bowel disease
